# Supplementary material for: High-speed optical imaging with sCMOS pixel reassignment
Source: Nat Commun. 2024 May 30;15:4598. doi: 10.1038/s41467-024-48987-7 (PMC11139943; doi:10.1038/s41467-024-48987-7)
Supplement: Supplementary file 1 — Supplementary Information, High-Speed Optical Imaging with sCMOS Pixel Reassignment [file 41467_2024_48987_MOESM1_ESM.pdf]

SUPPLEMENTARY INFORMATION

High-Speed Optical Imaging with sCMOS Pixel Reassignment

Biagio Mandracchia[1,2,†], Corey Zheng[1,†], Suraj Rajendran[1], Wenhao Liu[1], Parvin Forghani[3], Chunhui Xu[3,4], and Shu Jia[1,4,*]

[1]*Wallace H. Coulter Department of Biomedical Engineering, Georgia Institute of Technology and Emory University, Atlanta, GA, USA*

[2]*E.T.S.I. Telecomunicación, Universidad de Valladolid, Valladolid, Spain*

[3]*Department of Pediatrics, School of Medicine, Emory University, Atlanta, GA, USA*

[4]*Parker H. Petit Institute for Bioengineering and Bioscience, Georgia Institute of Technology, Atlanta, GA, USA*

[*] *shu.jia@gatech.edu*

SUPPLEMENTARY INFORMATION

Table of Contents

1 Supplementary Figures ... 3
2 System ... 15
2.1 Fiber bundle design ... 15
2.2 Fiber mapping ... 15
2.3 System integration discussion ... 15
2.4 Theoretical bounds of framerate improvement ... 16
2.5 Discussion on configuration design and advances ... 18
2.6 Comparison with other approaches ... 20
2.7 Signal-to-noise analysis ... 20
3 High-speed imaging flow cytometry ... 22
3.1 Stroboscopic illumination ... 22
3.2 Cell tracking ... 22
3.3 Theoretical maximum throughput ... 23
3.4 Analytical throughput ... 23
4 Cardiomyocyte trace analysis ... 24
5 Neuron calcium wave imaging ... 25
5.1 Excitation probe design ... 25
5.2 Isochronal map post-processing ... 25

1 Supplementary Figures

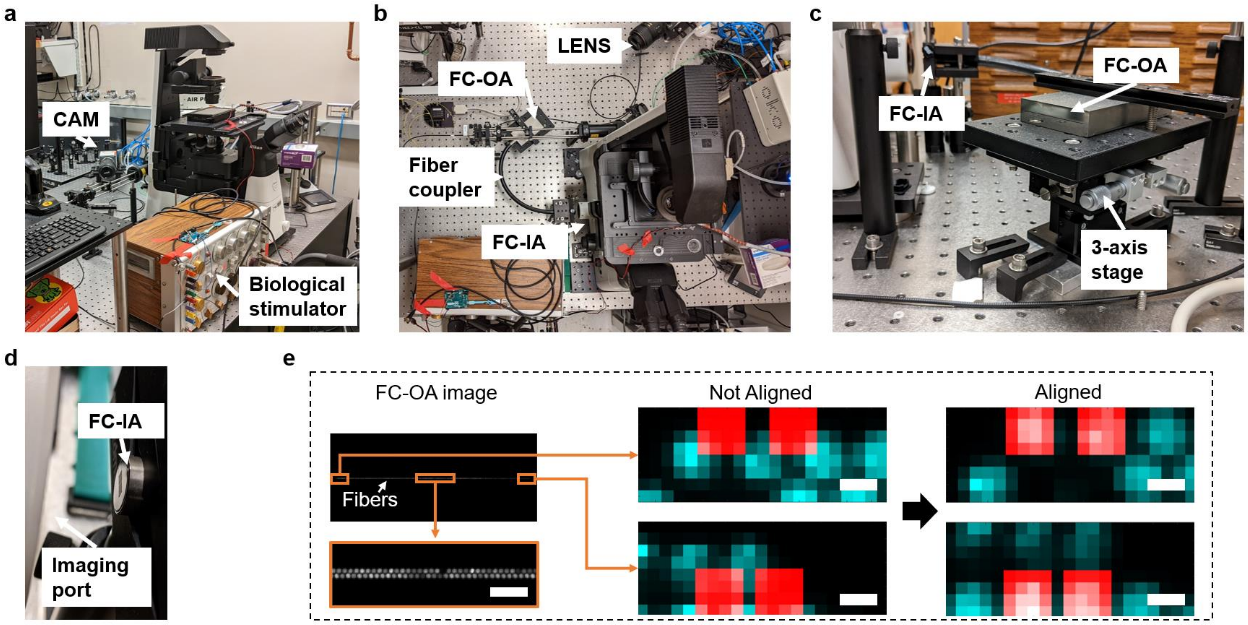

Supplementary Figure S1.

Images of physical system implementation and alignment process.

FC-IA, fiber coupler input array; FC-OA, fiber coupler output array. **(a)** Side view of the experimental setup. **(b)** Overhead view of experimental setup depicting fiber coupler positioning. **(c)** Close-up view of FC-OA mounting assembly on 3-axis stage for alignment. **(d)** Close-up view of FC-IA positioned at microscope imaging port. **(e)** Alignment process. A broadband white lamp on the microscope is used to illuminate the fiber coupler. Left: Raw image of FC-OA linear fiber array. Scale bar: 1 mm. Middle: Calibration software view of left-most (top) and right-most (bottom) fibers on the FC-OA along with 4×4 pixels red position markers indicating where the fibers should be located. Scale bar: 100 μm. Right: Alignment software view of fibers now in alignment with positional markers. Scale bar: 100 μm.

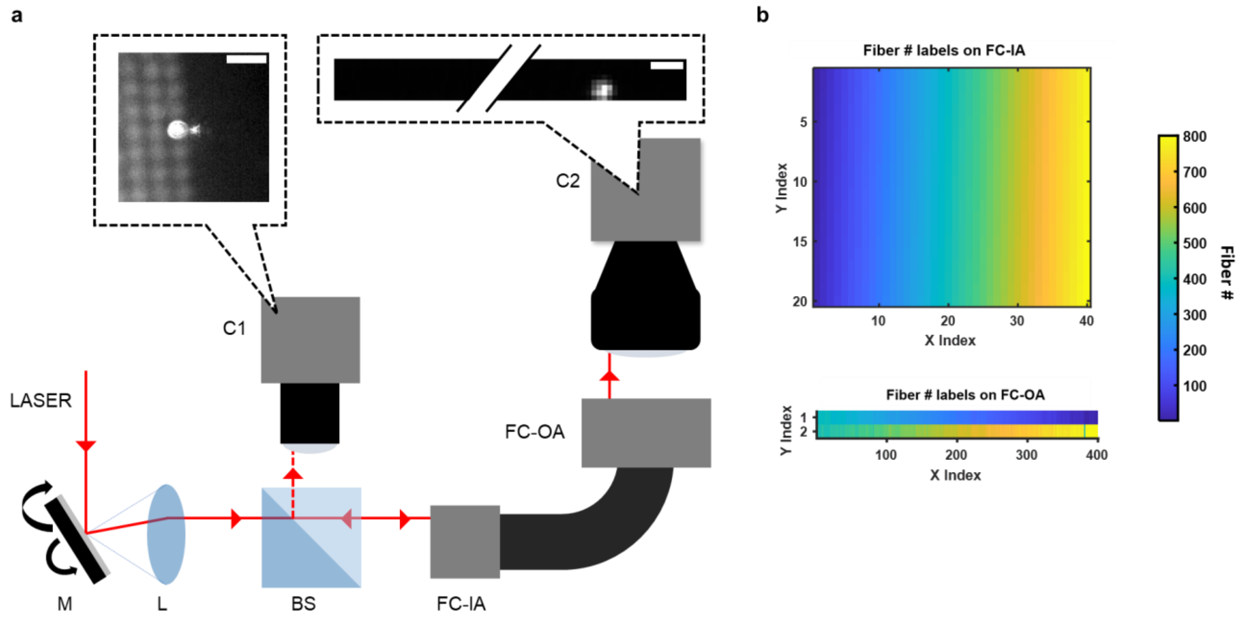

Supplementary Figure S2.

Diagram of fiber mapping setup and resultant fiber mapping indices.

(a) Fiber mapping measurement scheme. M, mirror; L, lens; BS, beam splitter; C, camera; FC-IA, fiber coupler input array; FC-OA, fiber coupler output array. Left callout, an image of fiber on the FC-IA illuminated by the laser, captured by C1. Scale bar: 0.3 mm. Right callout, an image of corresponding illuminated fiber ending on FC-OA captured by C2. Scale bar: 0.2 mm. **(b)** Resultant positions of corresponding fibers on the FC-IA and FC-OA as measured by the measurement scheme. Mapping is saved and used in all reconstructions.

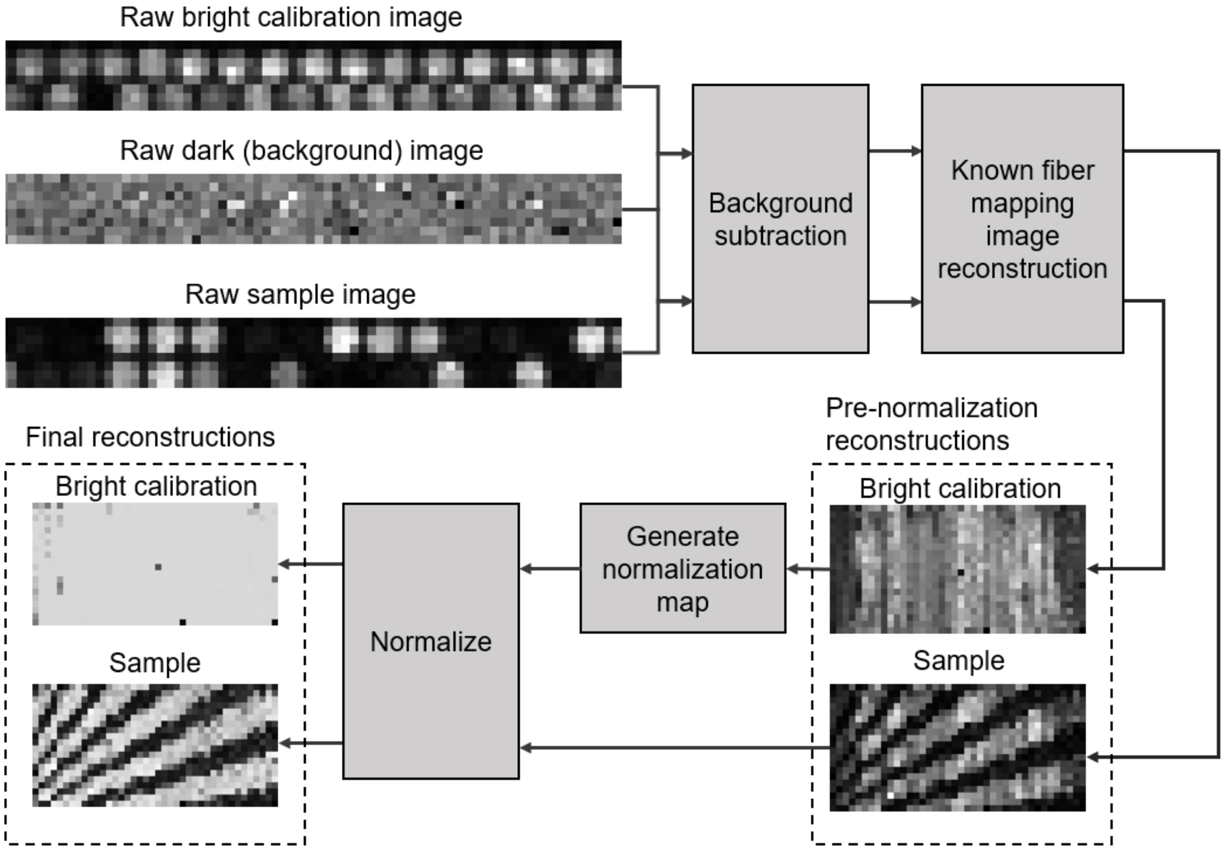

Supplementary Figure S3.

Universal sHAPR image reconstruction and processing scheme.

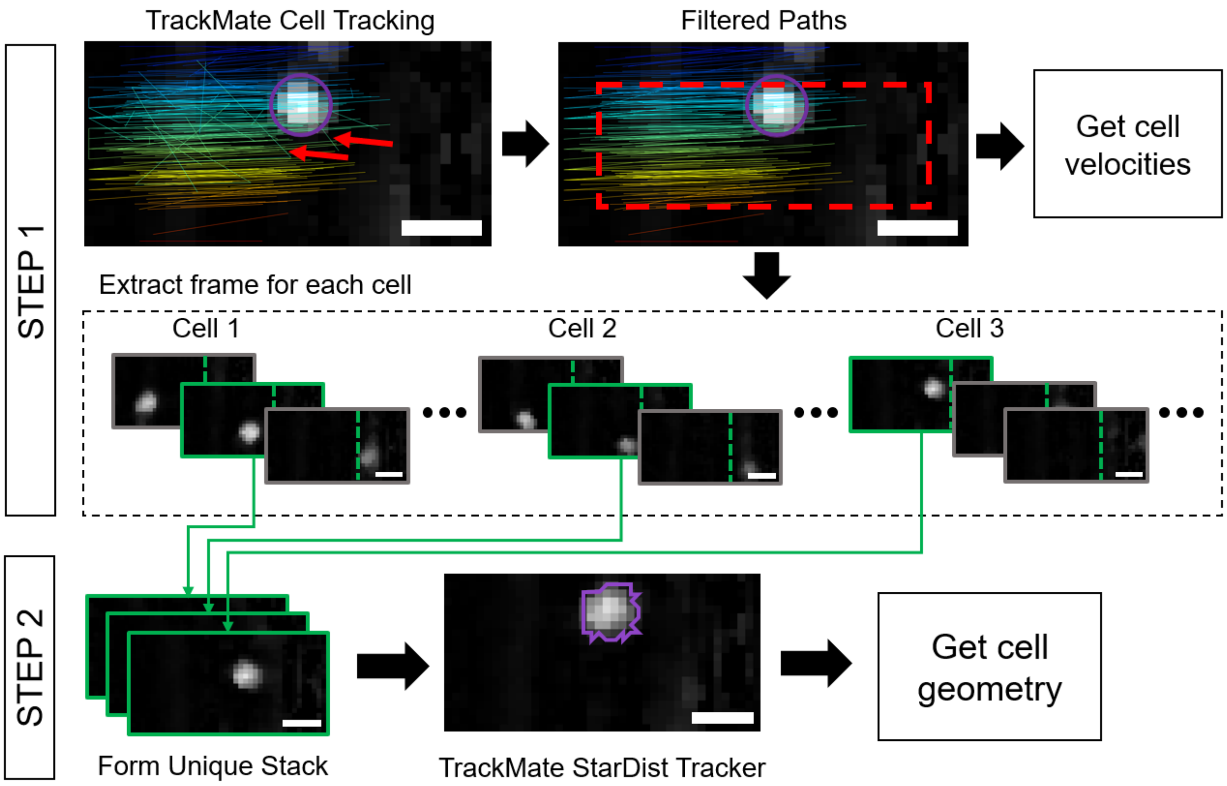

Supplementary Figure S4.

Detailed scheme for cell tracking in flow cytometry.

Scale bars: 20 μm.

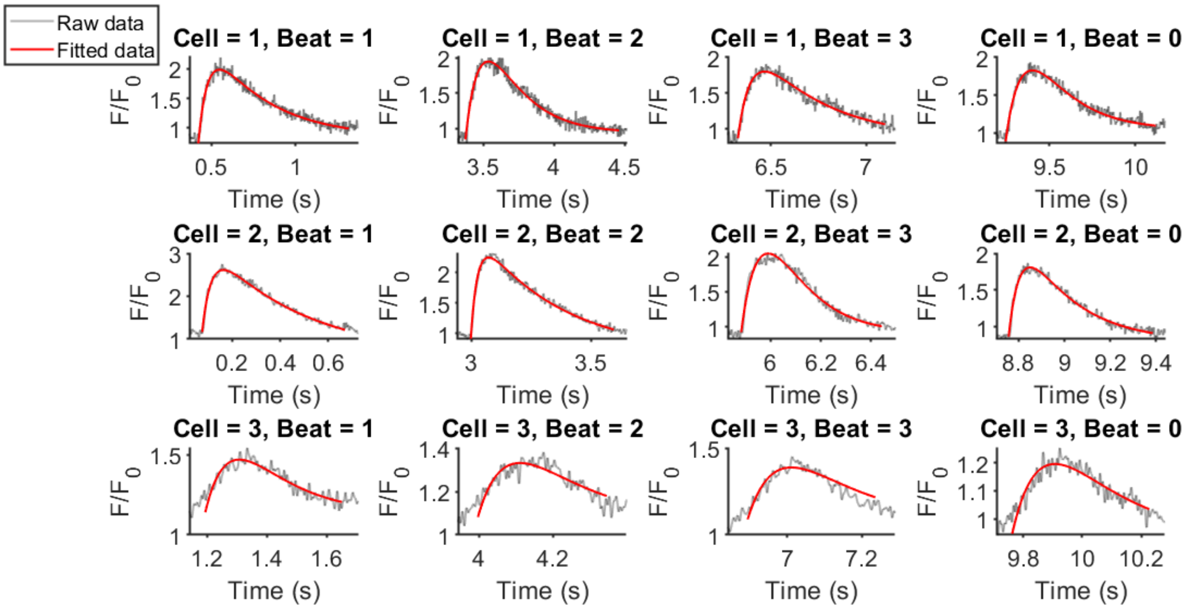

Supplementary Figure S5.

Additional fluorescent cardiomyocyte traces and fittings.

Data is shown across 3 cells with 4 excitation events per cell. Source data are provided as a Source Data file.

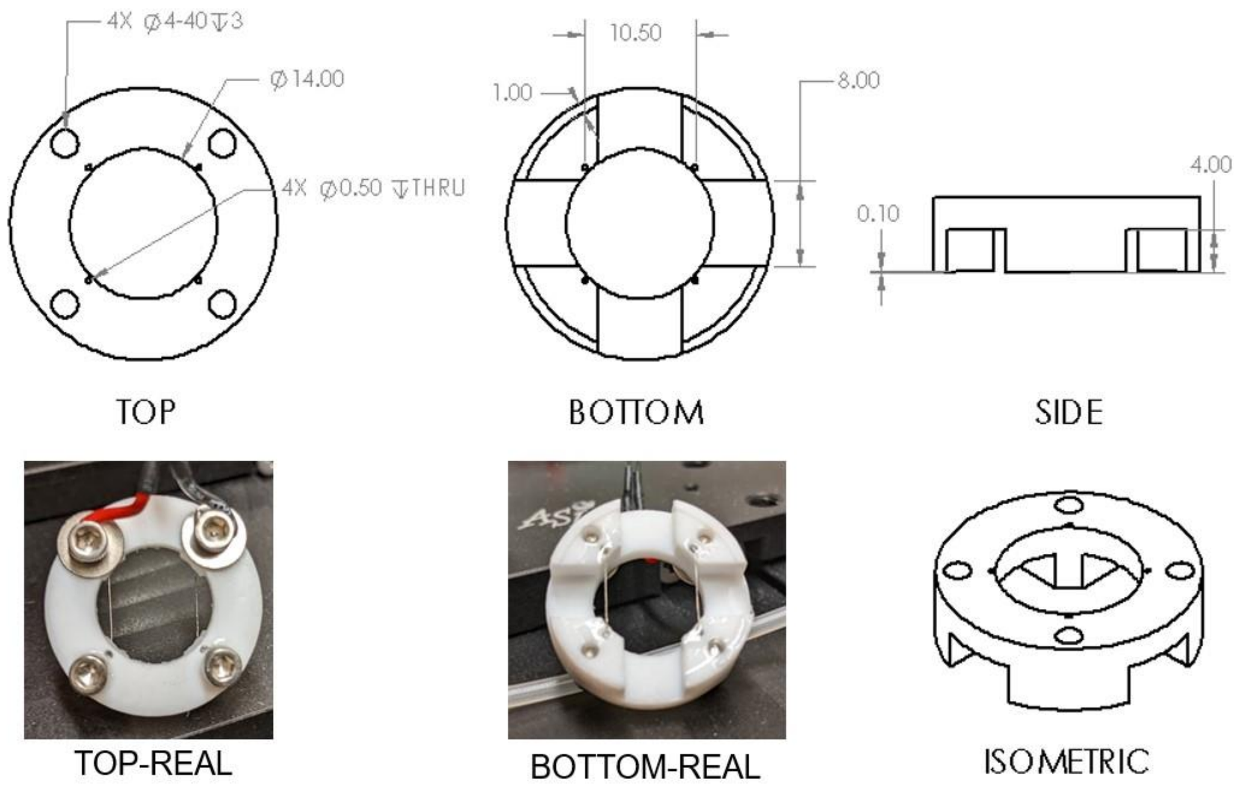

Supplementary Figure S6.

Neuron field stimulation probe.

Top left-top right, detail schematics and dimensions of the probe body. Bottom left, fabricated probe top-down view. Bottom middle, fabricated probe bottom-up view. Bottom right, isometric view of probe body design.

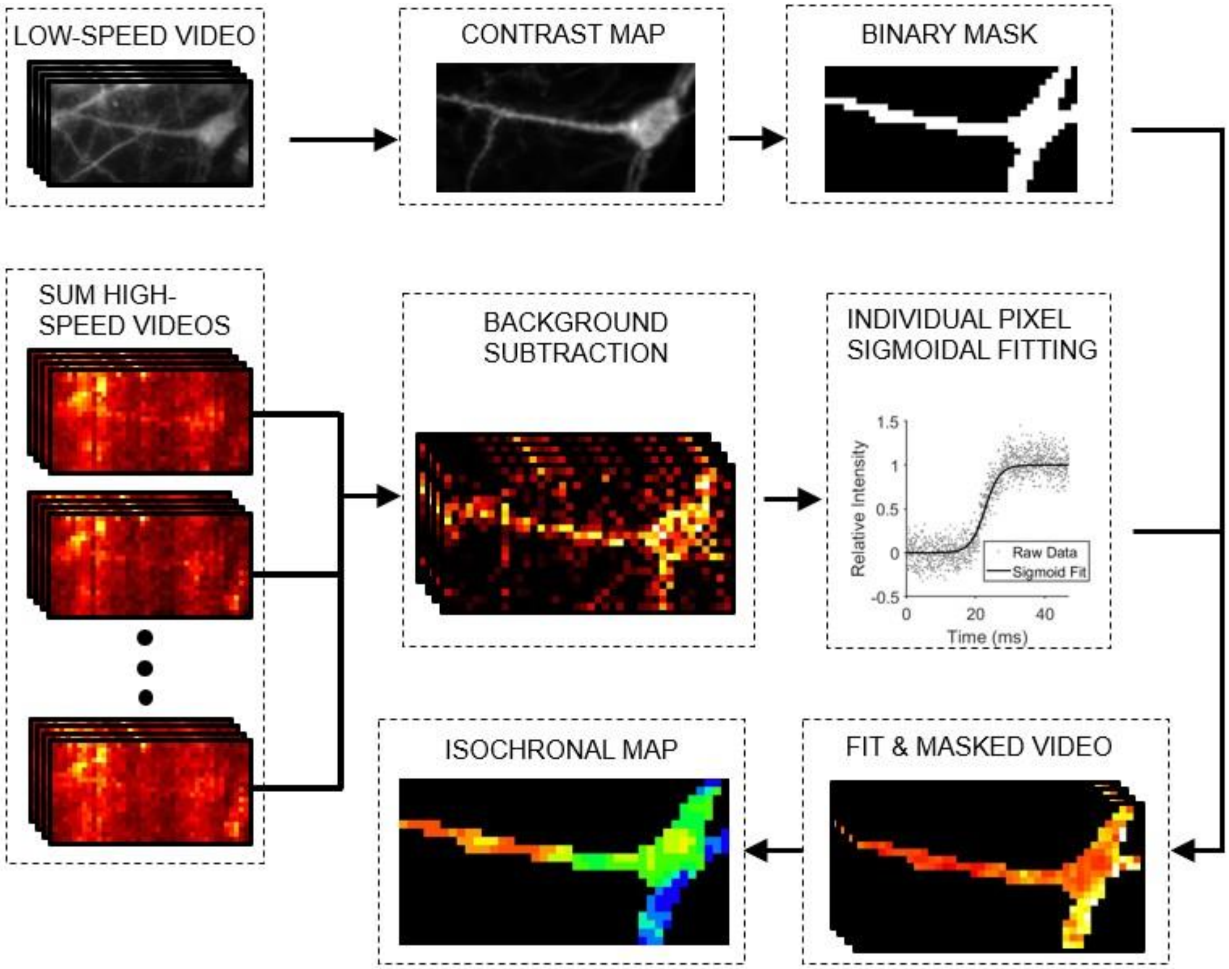

Supplementary Figure S7.

Post-processing procedure of neuron experiment.

Top row: A low-speed video of the neuron excitation is taken, and a contrast map is generated by subtracting the baseline image from a frame containing the excited neuron. The contrast map is then thresholded to generate a binary mask downscaled to the sHAPR image size. Middle row: Multiple excitations of the same neuron are captured and summed together. After subtracting the background signal, individual pixels are fit to sigmoidal curves. Bottom row: The pixels fitted to a sigmoidal function are masked to the shape of the neuron generated from the low-speed contrast map. Finally, an inverse sigmoid is used to solve for t_{p50}, and the results are binned to form the isochronal map.

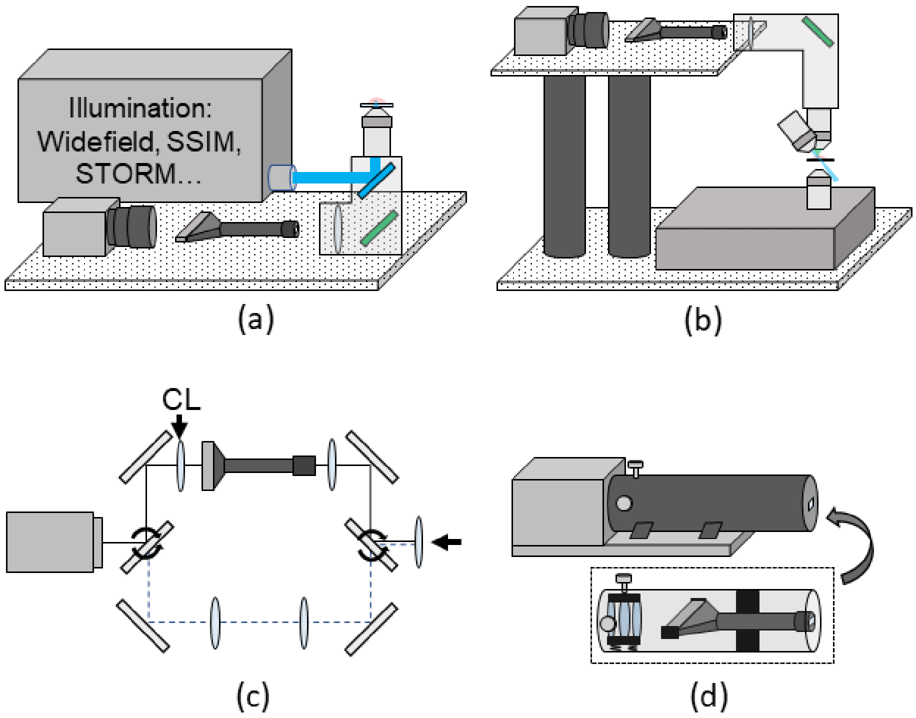

Supplementary Figure S8.

Possible integration schemes highlighting the adaptability of sHAPR.

(a) Detector-side sHAPR combined with arbitrary illumination schemes. **(b)** sHAPR integration with complex systems (diSPIM) as a bare CMOS replacement. **(c)** Fast switching between standard and sHAPR imaging modules. CL, coupling lens. (d) Possible drop-in sHAPR module with integrated optics.

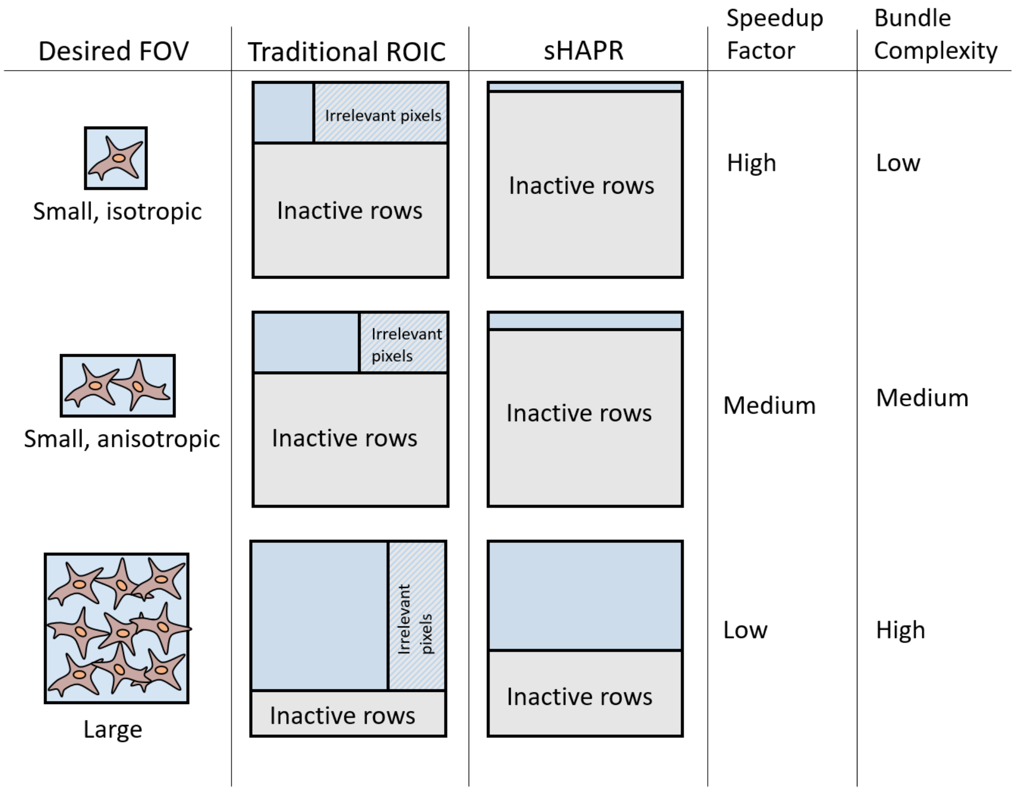

Supplementary Figure S9.

Comparison between traditional camera cropping and sHAPR.

Examples shown for different desired fields of view, assuming a fiber bundle fully utilizing the active pixel region. Solid blue: active pixels that are capturing the desired FOV. Dashed blue: pixels that can be read out with no speed penalty using traditional ROIC but are outside the desired FOV. Solid grey: inactive CMOS pixel rows.

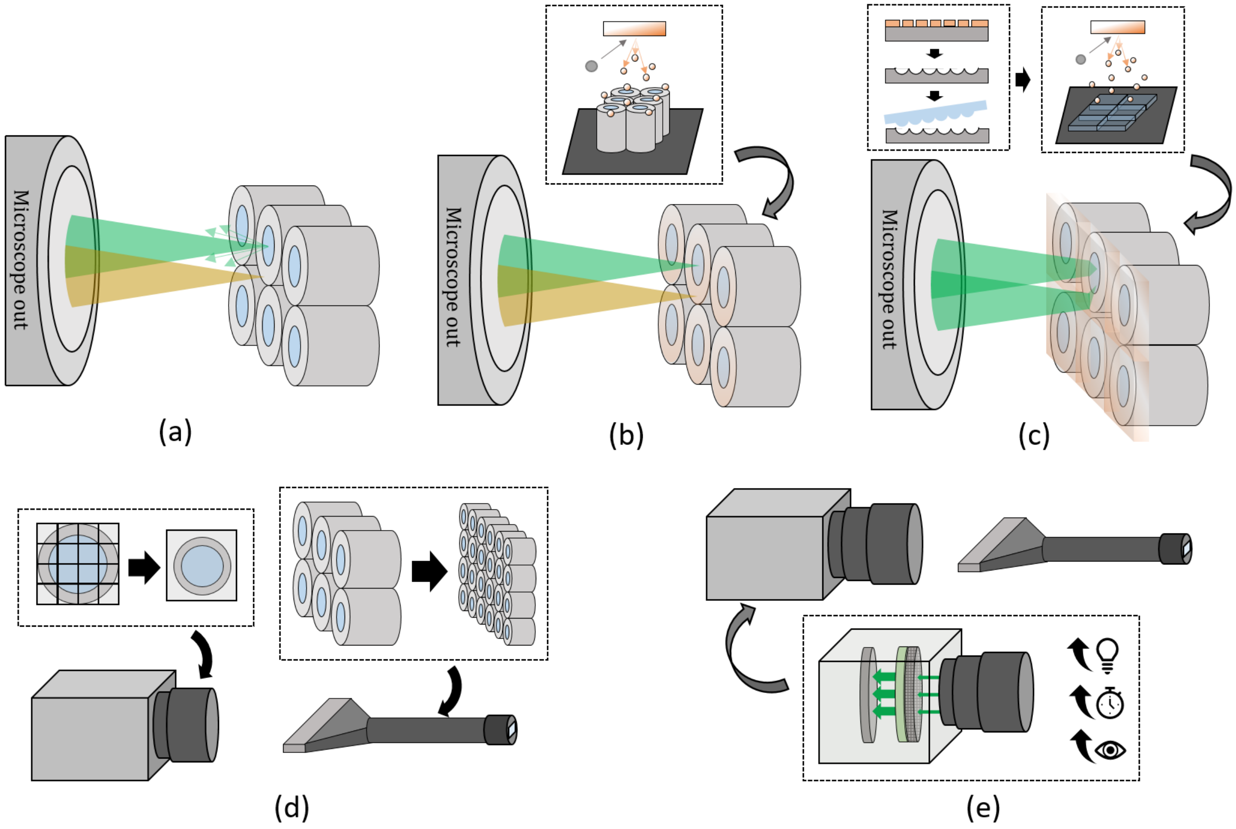

Supplementary Figure S10.

Examples of possible sHAPR upgrades.

(a) The current fiber bundle exhibits reflective loss and fill factor loss (yellow rays fall outside the core). **(b)** Anti-reflection coating can be applied through deposition processes (inset) and mitigates the reflective loss. **(c)** Micro-lens arrays can be fabricated through lithography (left inset) or purchased, anti-reflection coated (right inset), and bonded to fiber array to mitigate fill factor loss. **(d)** Smaller, densely packed fibers (right inset) with reduced coupling lens magnification (left inset) maximize pixel FOV. **(e)** Next-generation CMOS enables higher signal, imaging speed, and available pixels. Inset: intensified CMOS.

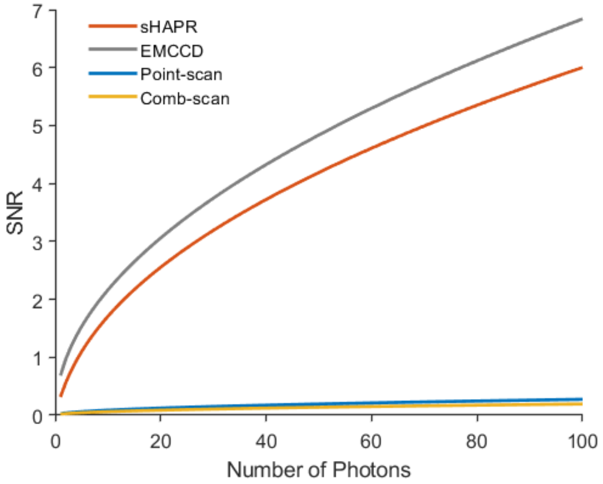

Supplementary Figure S11.

Theoretical SNR yielded by different high-speed imaging techniques.

Here, we plotted the SNR response of an EMCCD (gray), sHAPR (red), point-scan (blue), and comb-scan (yellow) according to the equations reported in Supplementary Section 2.7. For quantum efficiency, we considered 93% for the EMCCD, 37% for sHAPR, and 30% for both point- and comb-scan methods (as previously reported[1-3]). Moreover, for scanning techniques, we considered a scanning area of 20×20 pixels (bigger areas yield lower SNR). Finally, for the comb scan, we considered a uniform intensity distribution.

Table S1: Comparison of use-case scenarios in common applications for which isotropic FOV is required or anisotropic FOV can be beneficial.

<table>
<tr><th></th><th>Isotropic FOV</th><th>Anisotropic FOV</th></tr>
<tr><td>SPIM</td><td>Orthogonal Illumination (e.g., LSM)
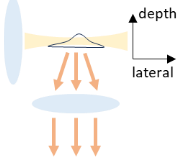

The sample is expected to extend equally on both lateral dimensions. The optical resolution is the same in both directions and isotropic sampling is recommended.</td><td>Oblique Illumination (e.g., SCAPE)
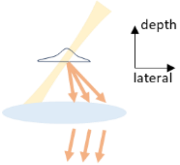

One of the image dimensions corresponds approximately to the sample depth and is expected to have less extent than the other.</td></tr>
<tr><td>Scan Imaging</td><td>Flow Cytometry
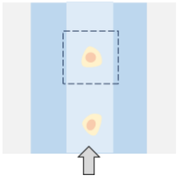
Flow control with micrometric precision during the whole measurement is hard to obtain. Throughput is normally limited by both camera speed and FOV in the flow direction. A small number of pixels in the flow direction makes the system prone to motion artifacts and limits the throughput. Still, a small number of pixels in the direction orthogonal to the flow reduces the image resolution considerably.</td><td>Industrial Inspection
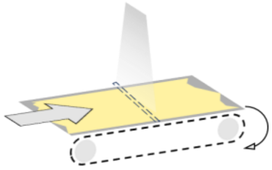
Scan speed can be controlled mechanically with high precision. Image resolution is limited by sampling. Reducing pixels in the scan direction allows the addition of more pixels in the other direction, thereby improving resolution in a cost-efficient way. Throughput is generally limited by factors other than camera speed.</td></tr>
<tr><td>Tracking</td><td>Unconstrained Diffusion
(e.g., proteins on cell membrane)
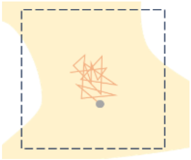
Diffusion is expected to be isotropic unless some phenomenon occurs. Motion direction is unknown even in the case of non-Brownian diffusion.</td><td>Hindered Diffusion
(e.g., proteins on filopodia)
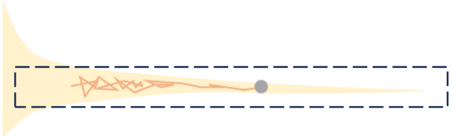
Diffusion is constrained by the morphology of the imaged region. Motion is practically limited to one direction.</td></tr>
<tr><td>Neuron Imaging</td><td>Soma & Dendrites Imaging
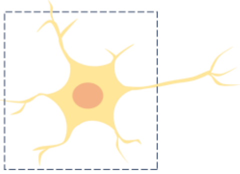
Signal propagation is expected to be isotropic. All the region around the soma is part of the area of interest.</td><td>Imaging of Single Axon
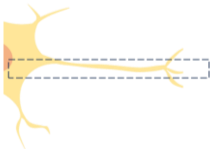
Only a single axon is monitored. Signal propagation is essentially one-dimensional.</td></tr>
</table>

2 System

2.1 Fiber bundle design

A custom-designed "Rectangle to Line" fiber bundle was purchased from Fiberoptics Technology Inc. The 0.5-m long bundle consists of 800 individual fibers arranged in a 20×40 rectangle on one side of the bundle and a 2×400 strip on the other end. Fibers are comprised of a pure fused silica core 100±3 μm in diameter, clad with fluorine-doped silica 110±3 μm in diameter, and finally coated with a polyimide coating, resulting in a total diameter of 125±3 μm per individual fiber. Fibers are composed of high OH silica and have a numerical aperture (NA) of 0.22±0.02. The pitch between fibers is 150 μm. Each end of the bundle was flat polished to a 0.3-μm tolerance. The fiber bundle and imaging system are shown in **Figure S1**. The bundle transmission was measured using a power sensor (Thorlabs S130C & PM100D) and determined to be 45% at 525nm. The fiber bundle was an incoherent bundle; thus, the exact correspondence of the fiber locations on each face was unknown and had to be measured as described in **Figure S2**. In total, the fiber bundle cost $4,275 and required an 8-week lead time; similar custom fiber arrays can be fulfilled by a variety of fiber optic manufacturers.

2.2 Fiber mapping

A mapping of the locations of corresponding fiber end-faces between the FC-IA and FC-OA was generated to properly reconstruct the sample image given an image of FC-OA. A schematic of the test setup used for this mapping is shown in **Figure S2a**. A hand-adjustable mirror M is mounted at the focal plane of lens L and is used to manually scan a laser beam across the face of the FC-IA. To determine which fiber on the FC-IA is being illuminated, the beam splitter BS is placed between L and FC-IA to direct reflected light into camera C1, as shown in the top left callout. Camera C2 images the FC-OA to capture the position of the other ending of the illuminated fiber, as shown in the top right callout. Despite slight flaws in the manufacturing process, the face of FC-IA is assumed to be a perfectly rectangular array, and each fiber is assigned an index based on its row and column position. As the fibers are illuminated one by one, the array index of each fiber on the FC-IA is recorded, along with its corresponding output pixel coordinate on the FC-OA image. Because the fibers occupy a 4×4 pixel region on the FC-OA image, we only record the index of the top-left corner pixel. The resultant mapping is illustrated in **Figure S2b**, and is used for all future reconstructions following the process shown in **Figure S3**.

2.3 System integration discussion

The integration of sHAPR in the detection path can drastically improve the imaging speed of virtually any standard sCMOS camera-based widefield system used for microscopy. Indeed, sHAPR is based on the rearrangement of two-dimensional images into monodimensional wavefronts. This is achieved entirely through passive optical waveguides without requiring any encoding of the incoming information. For this reason, the system can accept any bidimensional image independently from the sample illumination method employed. Thereby, sHAPR is compatible with both label-free and fluorescence imaging, including bright-field imaging, digital holography, total internal reflection mi-

croscopy, spinning disk confocal, selective plane illumination microscopy, tomographic phase microscopy, and Fourier ptychography. Interestingly, given the recent successful application of fiber relays to techniques such as single-molecule localization[4], structured illumination microscopy[5], and especially image scanning microscopy[6,7], we anticipate the possibility of sHAPR integration with these super-resolution techniques as well.

However, users must consider key tradeoffs such as fiber losses, binning, and spatial alignments to evaluate whether sHAPR will be suitable for their particular microscopy application. Below are several essential parameters to consider for successful integration.

Calibration and alignment. Correct calibration of the fiber mapping and precise alignment of the linear output from the fiber bundle with the camera's pixel array are imperative. Failure to achieve proper alignment may result in signal loss in specific pixels or signal crosstalk in more severe cases. To circumvent these issues, it is vital for users to ensure that the surface of the fiber output remains parallel to the surface of the CMOS sensor and that all fiber images are precisely focused and aligned with a single line of pixels. On the bright side, all fibers are glued together, so it is not necessary to check the alignment of all fibers. Instead, alignment checks can be generally restricted to only a few reference fibers at the extremes of the array.

Magnification. The pitch between fibers introduces a binning effect in the projection of the image from the microscope onto the fiber bundle, varying depending on the fiber size. Users may need to utilize a higher magnification compared to a standard camera approach when using sHAPR to achieve the desired sampling, which can be achieved by utilizing a higher magnification objective lens or introducing a magnifying relay between the fiber bundle and microscope.

Data acquisition. Optimizing data acquisition further necessitates positioning the fiber output at the center of the imaging sensor. This not only minimizes photo response non-uniformity[8] but, in many scientific cameras, also facilitates the utilization of twice the number of pixels, as many of these cameras offer the use of two adjacent sensors to enhance speed.

Light collection. Efficient light collection is paramount in high-speed imaging scenarios. Users are encouraged to fine-tune the coupling lenses to match the camera pixel size with the maximum value allowable for each experiment. Introducing a microlens array at the fiber input can also achieve better light collection and fill factor. Adding image intensifiers would provide further improvements for light-starved applications.

Data storage. Another challenge associated with high-speed imaging is the substantial volume of data generated, even when working with small ROIs. One potential approach to address this concern is to work with 8-bit images. While this lowers the camera's saturation threshold, this threshold is typically not encountered at high imaging speeds. Moreover, this adjustment can often increase the maximum achievable recording speed in many cases.

2.4 Theoretical bounds of framerate improvement

sHAPR operates on CMOS sensors utilizing column-parallel architectures, in which the framerate is proportional to the number of active pixel rows H rather than total number of pixels:

$$\text{FPS} \propto \frac{1}{H} \tag{1}$$

Rewritten, the imaging speed of a CMOS operating with subarray readout is dependent on the aspect ratio of the region $r = \frac{W}{H}$ and total number of pixels $N = H \times W$, where W is the number of active pixel columns.

$$\text{FPS(r}, N) \propto \sqrt{\frac{r}{N}} \tag{2}$$

The key advantage of sHAPR is that by reshaping the input image, it maximizes the effective aspect ratio at the camera plane (r) independently from the aspect ratio of the imaging FOV (r') so that $r > r'$ unless we use the entire CMOS sensor. Assuming a 1-to-1 mapping of points on the imaging plane to pixels on the camera plane and no gaps between fibers, this implies that the sHAPR method will always be faster than naïve sensor cropping as long as the desired FOV does not fully occupy the camera sensor in either dimension. The acceleration factor f of sHAPR can be calculated:

$$f = \frac{\text{FPS}_{\text{sHAPR}}(r, N)}{\text{FPS}_{\text{ROIC}}(r', N)} = \sqrt{\frac{r}{r'}} \tag{3}$$

It is clear that sHAPR provides the most benefit when an isotropic FOV is to be imaged (r' = 1) onto a highly anisotropic sensor region (r >> 1) (**Figure S9**).

However, the current implementation undersamples the imaging plane due to the binning effect of the fibers in the bundle, in which a single fiber is projected to a 4×4 pixel region. Thus, the number of pixels used in the camera sensor N is related to the number of fibers N_f by:

$$N = N_f \cdot p^2 \tag{4}$$

where p is the side length of the isotropic projection region in pixels. The acceleration factor is now:

$$f = \frac{\text{FPS}_{\text{sHAPR}}(r, N)}{\text{FPS}_{\text{ROIC}}(r', N)} = \frac{1}{p}\sqrt{\frac{r}{r'}} \tag{5}$$

This introduces a new condition. In order to enable a gain in imaging speed, $f > 1$, sHAPR must be constrained to a number of fibers less than:

$$N_f < \frac{W^2}{p^4 r'} \tag{6}$$

where $r' \cdot N_f \geq 1$. For example, for our current implementation where $p = 4$ and $W = 2048$, an isotropic ($r' = 1$) imaging FOV must be smaller than 128×128 fibers to be advantageous compared to standard ROIC.

2.5 Discussion on configuration design and advances

sHAPR can be readily upgraded with modern hardware. Generally, there are three main areas we foresee for future hardware advances, summarized in Figure S10: (1) Fiber bundle format, (2) Fiber bundle light collection optics, and (3) Camera hardware.

Regarding the fiber bundle format, the current 800-fiber bundle, mapping to 4×4 pixel regions, occupies 80% of active pixels. Enhancements may first involve increasing fiber count for full sensor coverage. Further reducing fiber-to-pixel projection by adjusting coupling lens magnification and increasing fiber count can enhance the field of view (FOV) or resolution for the same given sensor area (**Figure S10d**). The ultimate limit to this strategy would be a 1-to-1 fiber-to-pixel projection, enabling framerates on the order of 200kHz or higher with FOVs greater than 128×128 pixels depending on the camera model, explored in **Table S2**. Fiber-optic manufacturing companies are capable of custom fiber bundles in such large formats (e.g., 128×128 to 8×2048), and similar size arrays could also be hand-fabricated in the lab[9]. Additionally, the shape of the input array may be varied to achieve FOVs with varying anisotropy. Of course, fiber formats should also be selected with the camera hardware in mind, as other sensors may have different minimum row restrictions and pixel counts.

Fiber bundle light collection optics are crucial for increasing signal. Anti-reflection coatings can be applied onto fiber surfaces with deposition processes (**Figure S10b**) to decrease reflective loss by a few percent per surface (~2-4%). Furthermore, the fiber bundle fill factor is a major contributor to light loss, as only the core of the fiber bundle is receptive to light. This can be mitigated through the inclusion of a microlens array on the input side of the fiber bundle (**Figure S10c**), reflecting a similar approach taken in CMOS cameras to overcome pixel fill-factor issues[10].

Finally, we can leverage new cameras that offer combinations of extraordinary speed, high sensitivity, high quantum efficiency, and large imaging arrays (**Table S2**). Specialized options, such as new intensified sCMOS cameras with embedded image intensifiers, can enhance low-light performance (**Figure S10e**). The compatibility of sHAPR extends even to EMCCD cameras for burst imaging, utilizing their charge-shifting readout system in which rows are still exposed during the readout itself.

sHAPR provides a highly customizable approach, granting users a number of degrees of freedom in design. However, considerations for hardware compatibility are crucial. Matching fiber bundle output array shape to camera aspect ratios, accounting for different software limits, and adapting to varying pixel sizes with appropriate magnifying lenses are essential factors for optimal performance.

Table S2: Potential features of sHAPR when combined with state-of-the-art CMOS or CCD imagers.

Camera	Peak QE	Readout Noise	Active area	Fiber projection	FPS	Achievable sHAPR FOV	Required Fibers	Speed gain vs equal cropped FOV
Hamamatsu ORCA-flash4.0 v2 C11440 – *This work*	82%	$1.6e^-$	8×2048	4×4	25.6kHz**	20×40	800	2.5×
			8×2048	4×4	25.6kHz**	32×32	1024	4×
			8×2048	1×1*	25.6kHz**	128×128	16,384	16×
Teledyne Ki-netix sCMOS (“Sensitivity” mode)	93%	$1.2e^-$	8×3200	4×4	35.4kHz	40×40	1600	5×
			1×3200	1×1	283.1kHz	50×64	3200	50×
			8×3200	1×1	$35.4kHz^-$	160×160	25,600	20×
Teledyne Ki-netix sCMOS (“Speed” mode)	93%	$2.0e^-$	8×3200	4×4	200kHz	40×40	1600	5×
			1×3200	1×1	1.6MHz	50×64	3200	50×
			8×3200	1×1	200kHz	160×160	25,600	20×
HiCAM Fluo 2000	50%	$24e^-$***	4×1920	4×4	243kHz	20×24	480	5×
			64×1920	4×4	33.5kHz	80×96	7680	1.25×
			4×1920	1×1	243kHz	80×96	7680	20×
Andor iXon 888 Life EMCCD	95%	$<1.0e^-$***	1x1024	1×1	9.69kHz	32×32	1024	1×
			1024× 1024†	1×1	1.6MHz†	32×32	1024	N/A

* Fibers are currently projected to a 4×4 pixel region, but future implementations could be used to project fibers onto a single pixel.

** The ROI of the camera is at the minimum allowed by camera software; the framerate is at a maximum for this particular camera model.

*** Amplified cameras with adjustable gain levels

† This framerate is achievable based on the parallel-shifting or vertical clock functionality of the CCD camera, in which, during readout, the change on every pixel is shifted down by one row whilst still being exposed. This shift speed can be as low as 600ns, thus enabling a high-speed burst until all rows are read off the full sensor (1024 ‘frames’).

2.6 Comparison with other approaches

A summary of other major high-speed fluorescence microscopy approaches is tabulated in **Table S3**.

Table S3. Comparison of other high-speed fluorescence imaging methods.

Reference	Sampling (µm)	FOV (pix-els)	Spatial framer-ate	Image distor-tion	Imaging Category	Detector	Complex-ity	Cost	Compati-bility
**sHAPR (this work)**	1.0	20×40	25.6kHz	No	Widefield	sCMOS	Low	$	High
**Diebold et. al. - FIRE[1]**	0.47 (x), >0.47 (y) †	256×256	4.4kHz	Yes	Comb-scan	PMT	Very High	$$$	Low
**Wu et. al. - FACED[3,11,12]**	0.82 (x), 0.35 (y)	80×1200	3kHz	Yes	Point scan	PMT	Moderate	$$	Low
**Voleti et. al. - SCAPE 2.0[13]**	1.41 (x) 1.07 (y) ‡ 0.86 (z)	640×148	12.7kHz	No	Lightsheet scan	High-speed Intensified sCMOS	High	$$	Moderate
**Mikami et. al. – FDM confocal[14]**	0.21 (x) 0.18 (y)	190×231	16kHz	Yes	Comb-scan	PMT	Very High	$$$	Low
**Popovic et. al.[15]**	4	12×80	10kHz	No	Widefield	High-speed CCD	Low	$	High

† In the scanning direction, sampling is not constant because of the sinusoidal deflection pattern of the resonant mirror. Notably, the sine correction algorithm used to remove such distortion significantly lowers image resolution.
‡ The authors perform oblique scanning, which requires de-skewing of the acquired volume to translate the image from the camera coordinate system to the global coordinate system.

2.7 Signal-to-noise analysis

We offer a simplified signal-to-noise analysis to compare sHAPR with other imaging approaches, acknowledging that a comprehensive comparative analysis will necessitate careful consideration of specific design factors, including system losses, detector type, quantum efficiencies, dark current, and noise factors. As an initial assessment, sHAPR maximizes the signal-to-noise ratio (SNR) by employing high-quality sCMOS cameras. When operated with short exposure times or in a cooled low-noise regime, dark and spurious noise becomes negligible, making readout and shot noise the primary sources of noise. Consequently, the SNR expression can be simplified to:

$$\mathrm{SNR}_{\mathrm{sCMOS}}(p) = \frac{Q_E I_p}{\sqrt{Q_E I_p + R^2}} \tag{7}$$

where I_p is the number of photons incident on detector element p over the integration time, Q_E is the detector element quantum efficiency, and R represents the readout noise. Nowadays, the readout component for sCMOS cameras is typically $R \sim 1e^-$ or less due to recent industry advancements[16],

whilst the quantum efficiency is typically >80%. Factoring in our current fiber bundle losses (55%), the combined efficiency of sHAPR is approximately $Q_E \approx 0.37$. Comparatively, EMCCD cameras exhibit negligible readout noise but are generally slower and introduce noise associated with the amplification process, represented by the noise factor F:

$$\mathrm{SNR}_{\mathrm{EMCCD}}(p) = \frac{Q_E I_p}{\sqrt{F^2 Q_E I_p}} \tag{8}$$

where $F^2 \approx 2$ and $Q_E \geq 0.9$. Both sCMOS and EMCCD cameras capture pixels in parallel, maximizing exposure time for all pixels and increasing the overall SNR compared to serial acquisition methods like point-scanning confocal systems, which require dividing the total SNR by the number of pixels:

$$\mathrm{SNR}_{\text{point-scan}}(p) = \frac{Q_E I_p}{\sqrt{W \cdot H \cdot Q_E I_p}} \tag{9}$$

where W and H are the numbers of pixel columns and rows, respectively. Comb-scan methods partially address the limitations of serial point-scanning by recording pixel rows in parallel, increasing both imaging speed and integration time. However, frequency domain pixel mapping introduces shot noise cross-talk:

$$\mathrm{SNR}_{\text{comb-scan}}(p) = \frac{Q_E I_p}{\sqrt{2 \cdot H \cdot Q_E I_T}} \tag{10}$$

where I_T is the mean of the total number of photons collected in the scan line[1]. Thus, the shot noise measured from a particular pixel depends on how many photons are measured at all the other pixels in the line, so a set of bright pixels in a line scan will lead to more uncertainty in the measurement of all the other pixels, no matter their brightness. The quantum efficiency of the photomultiplier tubes (PMT) used in both scanning methods is $Q_E = 0.3$.

As shown by the equations above, high-speed imaging techniques must fundamentally trade off SNR for speed when compared to conventional sCMOS or EMCCD cameras. Thus, while high-speed scanning methods can achieve significantly higher frame rates than EMCCD cameras, they have to sacrifice photon efficiency, resulting in significantly reduced SNR. Interestingly, the sHAPR technique maintains superior effective quantum efficiency (photon collection) than both scanning approaches despite the light transmission loss inherent in the current implementation. Furthermore, being a widefield approach, sHAPR has a notable advantage over scanning methods by allowing simultaneous exposure across all pixels and avoiding channel cross-talk. Consequently, sHAPR can offer even faster frame rates than both point- and comb-scan techniques and also attain higher levels of SNR, which are closer to those of EMCCDs (**Figure S11**). Additionally, as discussed in **Supplementary Section 2.5**, optimizing the design of the fiber bundle holds promise for mitigating quantum efficiency losses, potentially reaching or even surpassing the SNR values of regular EMCCDs.

3 High-speed imaging flow cytometry

3.1 Stroboscopic illumination

In order to suppress motion blur for fast-moving cells, stroboscopic illumination was performed to reduce the effective camera exposure time, ideally constraining cell motion within a single exposure to less than one-half the pixel size. We used an acousto-optic tunable filter (AOTF) (PCAOM-VIS; Gooch & Housego) to shutter the laser. The AOTF oscillator is driven by an AOTF driver (97-03926-12; Gooch & Housego), which is controlled via external timing inputs produced by a function generator (33500B Trueform Waveform Generator; Keysight) to perform stroboscopic illumination. The function generator produces 1 V square pulses 5 μs in duration with an 8.2 ns ramp-up and ramp-down time at a frequency of 25,600 pulses per second to match the camera framerate. In order to achieve theoretically perfect motion blur suppression at a 2.5-μm pixel size (40× objective lens) and 1m/s flow speed, we would need an illumination duration of 1.25 μs. Unfortunately, the AOTF had inconsistent responses to shorter pulse duration, limiting our stroboscopic illumination to a minimum of 5 μs with the current setup. Despite this, we still achieve a major reduction in motion blur, and such illumination can be shortened further with alternative shuttering schemes or improved AOTF hardware.

3.2 Cell tracking

Cell analysis was performed using the ImageJ TrackMate plugin. Originally, we attempted to utilize the StarDist detector[17] to perform simultaneous tracking and cell morphology analysis on the full image sequence but were impeded by the long processing times required on our computing systems (~12 hrs for one 30,000 frame sequence containing several hundred cells). In order to resolve this, we devised a two-step scheme to individually perform cell tracking and morphological analysis, as depicted in **Figure S4**.

First, image stacks were processed to track cells, generating a set of valid cell paths that detailed the velocity and locations of individual cells across different frames. Second, using this tracking information, the first frame in which a unique cell body appears was extracted from the image stack to create a new sequence in which every unique cell identified by tracking appears in exactly one frame. This new stack is processed using the TrackMate StarDist detector for the accurate measurement of cell area and, correspondingly, cell diameter.

In step 1, image stacks of 30,000 frames each (1.16s in duration) were first processed in TrackMate using the simple LoG detector scheme with an estimated diameter of 15μm. With this tracking option, the recorded cell diameter is set equal to the user's initial estimate of the cell diameter, necessitating a second step to measure the cell geometry. Median filtering was enabled. The quality threshold was empirically adjusted to detect only cells. The LAP tracking scheme was used, with a frame-to-frame linking distance equal to the maximum possible distance a cell could move within 1 frame (40 μm for a theoretical flow speed of 1 m/s), and gap closing was not allowed. To filter out erroneous traces, a combination of "linearity of forward progression" and "track displacement" filters was applied to retain cell paths that were mostly straight and horizontal, eliminating any diagonally oriented or very

short cell paths. A comma-separated value (CSV) file of resultant cell paths was exported and further filtered in MATLAB, in which any cells with a center that passed closer than 10 µm to the top and bottom of the image were discarded to ensure only fully in-frame cells were retained.

In the second step, a new image sequence containing a single frame of each unique cell was created by extracting the first frame in which each cell appears relatively centered in the image (beyond 15 pixels from the image border). TrackMate was used to process this new stack using the StarDist detector, and the result spot information containing the calculated area of each cell was used to calculate the resultant radius distribution under the assumption of a circular nucleus. This process was repeated for each experimental group.

3.3 Theoretical maximum throughput

The theoretical maximum flow that can be captured by the current system at 100× magnification is calculated as 1.5 m/s. At this magnification, the pixel size is 1.5 µm, and the resulting field of view (FOV) is 30×60 µm. At the maximum camera acquisition speed of 25,600 frames per second (FPS), the maximum flow v_{max} rate which can be fully sampled is calculated as:

$$v_{max} = \frac{\mathrm{FOV}_l}{1/\mathrm{FPS}} \tag{11}$$

where FOV_l is the length of the FOV along the direction of flow, and FPS is the camera framerate in Hz. For our system, this evaluates to a maximum achievable flow of 1.54 m/s, assuming that stroboscopic illumination of a short enough duration can be used to control the motion blur.

3.4 Analytical throughput

Based on the theoretical maximum flow described in Supplementary Section 3.3, we calculated the analytical throughput of our system under a number of assumptions. We assume a spacing d between cells of 40µm, that cells flow single-file into the FOV, and that we can achieve sufficiently short stroboscopic illumination to negate motion blur, which is approximately half a microsecond in all cases. Thus, the throughput c becomes:

$$c = \frac{\mathrm{FPS} * \mathrm{FOV}_l}{d} \tag{12}$$

The maximum flow speed and cell throughput for a variety of magnifications are calculated in **Table S4**.

Table S4. Comparison of analytical throughputs of current system given different magnifications.

Magnification	FOV (µm)	Maximum flow (m/s)	Required strobe duration (µs)	Throughput (cells/s)
20×	150×300	7.68	0.4883	192,000
30×	100×200	5.12	0.4883	128,000
40×	75×150	3.84	0.4883	96,000
60×	50×100	2.56	0.4883	64,000
100×	30×60	1.536	0.4883	38,400

4 Cardiomyocyte trace analysis

To extract relevant parameters from trace data, pCLAMP software was used. A fluorescent intensity time trace covering at least 4 distinct excitation-contraction events was extracted from a 3-by-3 region within each cell body (n = 3). Within pCLAMP, manual event detection was performed on each trace, and excitation curves were fitted using a product-of-two-exponentials function or sum-of-two-exponentials function. Calcium transients are controlled by a complex set of underlying physiological processes[18,19]. Generally, fluorescent activity produced by such transients in excitable cells (cardiomyocytes, neurons) has been modeled with mono-exponentials, sum-of-exponentials[20], half-logistic functions[21], sequential exponentials[19], product-of-exponentials[22], and more advanced functions incorporating cellular kinetics[18]. For this experiment, we found that the fluorescent activity was well-fit by two default provided functions: the product of two exponential functions and the sum of two standard exponentials (**Figure S5**), which resulted in fittings with correlation coefficients >0.80. The sum of two exponentials takes the form of:

$$f(t) = A_1 e^{\frac{-t}{\tau_r}} + A_2 e^{\frac{-t}{\tau_d}} + C \tag{13}$$

The product of two exponentials takes the form of:

$$f(t) = A\, e^{\frac{-t}{\tau_d}} \left(1 - e^{\frac{-t}{\tau_r}}\right) + C \tag{14}$$

We fitted each curve using both functions, selected the function that had the highest correlation coefficient, and extracted the relevant constants. We then imported these constant values into MATLAB, replicating the fitting function for each trace, and solved for relevant physiological parameters.

5 Neuron calcium wave imaging

5.1 Excitation probe design

A field excitation probe was constructed following the general design presented previously[23]. **Figure S6** shows a schematic of the probe. A 1-inch diameter PTFE rod (8546K16; McMaster Carr) was cut to produce a 7 mm thick disc. A central circle with an indentation of 0.1mm, to compensate for the thickness of the electrode wires, was milled into the disc. Then, two 8 mm wide × 4 mm deep rectangular channels in a cross pattern were milled into the disc, forming the 4 feet the probe stands on. A central hole 14 mm in diameter was drilled. Four small holes, arranged on the vertices of a 10.5 × 10.5 mm square, were drilled through the feet of the probe. Thin 0.005-in-diameter platinum wire (6205N26; McMaster Carr) was threaded through the holes to form two parallel electrodes. 4-40 screws were threaded into the top surface of the probe to clamp down the ends of the wire, providing tension and an electrical connection from the electrodes to the biological stimulator (S88 Biological Stimulator; Grass Instruments). PDMS (Sylgard 184; Dow Corning) was used to seal any holes. Prior to imaging, the entire probe was immersed in 70% isopropyl alcohol and allowed to dry.

5.2 Isochronal map post-processing

A flowchart of the post-processing procedure is shown in **Figure S7**. We found that excess imaging time induced cellular damage due to phototoxicity, resulting in neurons that would no longer respond to stimulation[24]. Because of this, we restricted laser illumination power to the 30mW setting. In combination with the low exposure time per frame during high-speed recording (38.97μs), we found that the recorded video had a low signal-to-noise ratio (SNR). To overcome this, we recorded at least 10 excitations for each candidate neuron using our high-speed system. Each video was 30,000 frames in length. We then summed the videos of all excitations of a given neuron, resulting in a video of a much higher signal.

We then performed post-processing in MATLAB. Data was first smoothed by applying a Gaussian filter (σ = 0.5), then a loess filter with a window of 20 samples on the time series of each pixel. Afterwards, to better show propagation in the neuron, we fit the intensity of the rising phase of a neuron to sigmoidal curves[25]. Each video was first cropped to 1,200 frames corresponding to the rising phase of the neuron activity, starting at the known time point of electrical stimulation. Each pixel time series was then non-linearly fit using the MATLAB nlinfit function to a sigmoidal curve using the form:

$$I = \frac{m - b}{1 + e^{w(\alpha - t)}} + b \tag{15}$$

where I is pixel intensity, t is the timestamp of the frame, and m, b, w, and α are function parameters. m and b reflect the minimum and maximum intensity values the pixel reaches, respectively. After parameterizing the activity of each pixel in time with these four constants, m, and b were set to 1 and 0, respectively, to normalize each pixel.

To prevent inactive pixels outside of the neuron from also being fit and normalized, the image stacks were first masked. Masks were generated using the high-resolution low-speed video taken prior to high-speed acquisition. By subtracting images of the neuron's ”idle” and ”excited” states, a contrast map is generated, which is then denoised using the ACsN algorithm previously reported by our group[8] and downscaled to the size of the sHAPR system image. From this image, a binary mask of the neuron is created by tracing over the visible cell body, attempting to keep only the processes from the one or two cell bodies visible in the image.

Then, the inverse sigmoid function is used to solve for the time at which the pixel reaches 50% of its peak intensity, t_{p50}:

$$t_{p50} = -\frac{1}{w}\ln\left(\frac{m-b}{I-b}-1\right)+\alpha \qquad (16)$$

Constants w and α are retained from the sigmoidal fitting. Because we only care about the time at which the relative intensity reaches 50% of its maximum, the constants m, b, and I are fixed at $m = 1$, $b = 0$, $I = 0.5$. The result is a 2D image of timestamps corresponding to the time to 50% of the peak for each pixel. The resulting matrix is processed with a median filter of size 3, and the lower 5th and upper 95th percentile of times were discarded to remove outliers. Finally, the image was binned into 10 evenly-sized time bins and color-mapped, resulting in the final isochronal maps.

Supplementary References

1 Diebold, E. D., Buckley, B. W., Gossett, D. R. & Jalali, B. Digitally synthesized beat frequency multiplexing for sub-millisecond fluorescence microscopy. *Nature Photonics* **7**, 806-810 (2013). https://doi.org:10.1038/nphoton.2013.245

2 Hamamatsu. *Photomultiplier Tubes Basics and Applications*. 4 edn, (Hamamatsu Photonics K.K., 2017).

3 Wu, J. *et al.* Kilohertz two-photon fluorescence microscopy imaging of neural activity in vivo. *Nature Methods* **17**, 287-290 (2020).

4 Israel, Y., Tenne, R., Oron, D. & Silberberg, Y. Quantum correlation enhanced super-resolution localization microscopy enabled by a fibre bundle camera. *Nature Communications* **8**, 14786 (2017).

5 Bozinovic, N., Ventalon, C., Ford, T. & Mertz, J. Fluorescence endomicroscopy with structured illumination. *Optics Express* **16**, 8016-8025 (2008).

6 Tenne, R. *et al.* Super-resolution enhancement by quantum image scanning microscopy. *Nature Photonics* **13**, 116-122 (2019).

7 Huff, J., Bathe, W., Netz, R., Anhut, T. & Wieisshart, K. Technology Note: The Airyscan Detector from ZEISS Confocal Imaging with Improved Signal-to-Noise Ratio and Superresolution. (2015).

8 Mandracchia, B. *et al.* Fast and accurate sCMOS noise correction for fluorescence microscopy. *Nature Communications* **11**, 94 (2020).

9 Wang, Y., Pawlowski, M. E. & Tkaczyk, T. S. in *Imaging, Manipulation, and Analysis of Biomolecules, Cells, and Tissues IX.* 209-213 (SPIE).

10 Taylor, S. A. CCD and CMOS imaging array technologies: technology review. *UK: Xerox Research Centre Europe*, 1-14 (1998).

11 Wu, J.-L. *et al.* Ultrafast laser-scanning time-stretch imaging at visible wavelengths. *Light: Science & Applications* **6**, e16196-e16196 (2017).

12 Wu, J. *et al.* Multi-MHz laser-scanning single-cell fluorescence microscopy by spatiotemporally encoded virtual source array. *Biomedical Optics Express* **8**, 4160-4171 (2017).

13 Voleti, V. *et al.* Real-time volumetric microscopy of in vivo dynamics and large-scale samples with SCAPE 2.0. *Nat Methods* **16**, 1054-1062 (2019). https://doi.org:10.1038/s41592-019-0579-4

14 Mikami, H. *et al.* Ultrafast confocal fluorescence microscopy beyond the fluorescence lifetime limit. *Optica* **5**, 117-126 (2018).

15 Popovic, M. A., Foust, A. J., McCormick, D. A. & Zecevic, D. The spatio-temporal characteristics of action potential initiation in layer 5 pyramidal neurons: a voltage imaging study. *The Journal of physiology* **589**, 4167-4187 (2011).

16 Hamamatsu. Technical note: ORCA-Quest qCMOS camera C15550-20UP. (2022).

17 Schmidt, U., Weigert, M., Broaddus, C. & Myers, G. in *Medical Image Computing and Computer Assisted Intervention–MICCAI 2018: 21st International Conference, Granada, Spain, September 16-20, 2018, Proceedings, Part II 11.* 265-273 (Springer).

18 Zahradníková Jr, A., Poláková, E., Zahradník, I. & Zahradníková, A. Kinetics of calcium spikes in rat cardiac myocytes. *The Journal of Physiology* **578**, 677-691 (2007).

19 Lacampagne, A., Ward, C. W., Klein, M. G. & Schneider, M. F. Time course of individual Ca2+ sparks in frog skeletal muscle recorded at high time resolution. *The Journal of general physiology* **113**, 187-198 (1999).

20 LLC, I. & Palmer, B. (IonOptix LLC, Milton, MA, 2019).

21 Mizuno, J., Otsuji, M., Yokoyama, T., Arita, H. & Hanaoka, K. Half-logistic function model for first half of descending phase of cardiomyocyte cytoplasmic Ca2+ concentration ([Ca2+] i)-

time curve (CaTCIII) in isolated aequorin-injected mouse left ventricular papillary muscle. *Acta Cardiologica Sinica* **32**, 65 (2016).

22 Grewe, B. F., Langer, D., Kasper, H., Kampa, B. M. & Helmchen, F. High-speed in vivo calcium imaging reveals neuronal network activity with near-millisecond precision. *Nature Methods* **7**, 399-405 (2010).

23 Stern, S., Rotem, A., Burnishev, Y., Weinreb, E. & Moses, E. External excitation of neurons using electric and magnetic fields in one-and two-dimensional cultures. *JoVE (Journal of Visualized Experiments)*, e54357 (2017).

24 Icha, J., Weber, M., Waters, J. C. & Norden, C. Phototoxicity in live fluorescence microscopy, and how to avoid it. *BioEssays* **39**, 1700003 (2017).

25 Lipkin, A. M., Cunniff, M. M., Spratt, P. W., Lemke, S. M. & Bender, K. J. Functional microstructure of CaV-mediated calcium signaling in the axon initial segment. *Journal of Neuroscience* **41**, 3764-3776 (2021).
