# Supplementary material for: High-speed optical imaging with sCMOS pixel reassignment
Source: Nat Commun. 2024 May 30;15:4598. doi: 10.1038/s41467-024-48987-7 (PMC11139943; doi:10.1038/s41467-024-48987-7)
Supplement: Supplementary file 3 — Description of Additional Supplementary Files [file 41467_2024_48987_MOESM3_ESM.pdf]

Description of Additional Supplementary Files

Supplementary Movie 1: Brightfield imaging using sHAPR. First sequence: grains of sand suspended in water moving in a microfluidic channel. Second sequence: air bubble moving through a microfluidic channel. Scale bars: 10 µm.

Supplementary Movie 2: Imaging Flow Cytometry experiment. First sequence: cell flow at a theoretical channel flow speed of 0.2m/s at 40× and 100× magnification. Second sequence: cell flow at a theoretical channel flow speed of 1.0m/s at 40× and 100× magnification. Scale bar: 10 µm.

Supplementary Movie 3: Cardiomyocyte whole cell excitation-contraction cycle. First sequence: Brightfield image of singular excitation-contraction cycle. Second sequence: sHAPR 1kHz fluorescence imaging of four subsequent excitation-contraction cycles of the same region. Third sequence: Slow-motion playback of a single cycle acquired using sHAPR visualizing calcium wave propagation. The "6 shades" color map from ImageJ is used. This sequence is processed with a 2D smoothing Gaussian filter with a variance parameter of 1. Scale bars: 10 µm.

Supplementary Movie 4: Imaging of subcellular calcium sparks. First sequence: low-speed (30Hz) widefield fluorescent imaging of subcellular sparking activity occurring at different locations within the cell. Second sequence: High-speed sHAPR 9.35kHz imaging of subsequent activity within the same cell. Third sequence: Comparison between sHAPR and widefield sequences. sHAPR clearly resolves propagation of the central calcium wave cell that occurs within 1 frame of the widefield video. Scale bars: 10 µm.

Supplementary Movie 5: Imaging of calcium sparks coalescing into a full-cell excitation wave. First sequence: low-speed (30Hz) widefield fluorescent imaging of observed phenomena, starting with a full-cell wave, followed by rapid calcium sparks, followed by

another full-cell wave. Second sequence: High-speed sHAPR 9.35kHz imaging of subsequent activity within the same cell. Third sequence: Comparison between sHAPR and widefield sequences. As indicated in the red box, the propagation of a calcium wave initiated in the same region of the cell occurs within 1 frame of the widefield video but can be clearly resolved using high-speed sHAPR. Scale bars: 10 µm.

Supplementary Movie 6: Example of neuronal field stimulation. A green indicator shows the time of stimulation. Culture-wide neuron depolarization can be observed in response to an electrical field stimulation pulse. Scale bar: 50 µm.

Supplementary Movie 7: Depiction of captured neuron stimulation and of multiple averaged stimulations. First sequence: low-speed (100Hz) widefield raw fluorescence imaging of single stimulated neuron excitation. Second sequence: A single stimulation of the same neuron imaged using high-speed sHAPR (25.6kHz). Video is first filtered using a loess filter with a 20-sample window and is subsequently processed to display change in fluorescence (F/F_0). Third sequence: 40 stimulations of the same neurons imaged using high-speed sHAPR (25.6kHz), then averaged together and subject to the same processing as the second sequence. The resultant video is overlaid on a static widefield captured image. All scale bars: 15µm.

Supplementary Movie 8: Movie showing the rising phase of an AP-initiated calcium wave. First sequence: low-speed (100Hz) widefield raw fluorescence imaging of single stimulated neuron excitation. Second sequence: Visualization of active areas of the cell by creating a fluorescent contrast map before and after stimulation, which is subsequently down-sampled and used to mask out background signals. Third sequence: Rising phase AP initiated calcium wave. The movie was created from ≥10 averaged acquisitions, and each pixel was subject to fitting as described in Supplementary Section 5.2. All scale bars: 15 µm.

Supplementary Movie 9: Movie showing the rising phase of an AP-initiated calcium wave. First sequence: low-speed (100Hz) widefield raw fluorescence imaging of single stimulated neuron excitation. Second sequence: Visualization of active areas of the cell by creating a fluorescent contrast map before and after stimulation, which is subsequently down-sampled and used to mask out background signals. Third sequence: Rising phase AP initiated calcium wave. The movie was created from ≥10 averaged acquisitions, and each pixel was subject to fitting as described in Supplementary Section 5.2. All scale bars: 15 µm.

Supplementary Movie 10: Movie showing the rising phase of an AP-initiated calcium wave. First sequence: low-speed (100Hz) widefield raw fluorescence imaging of single stimulated neuron excitation. Second sequence: Visualization of active areas of the cell by creating a fluorescent contrast map before and after stimulation, which is subsequently down-sampled and used to mask out background signals. Third sequence: Rising phase AP initiated calcium wave. The movie was created from ≥10 averaged acquisitions, and each pixel was subject to fitting as described in Supplementary Section 5.2. All scale bars: 10 µm.

Supplementary Software: The core package for sHAPR
