# Supplementary figures and images for: High-speed optical imaging with sCMOS pixel reassignment
Source: Nat Commun. 2024 May 30;15:4598. doi: 10.1038/s41467-024-48987-7 (PMC11139943; doi:10.1038/s41467-024-48987-7)

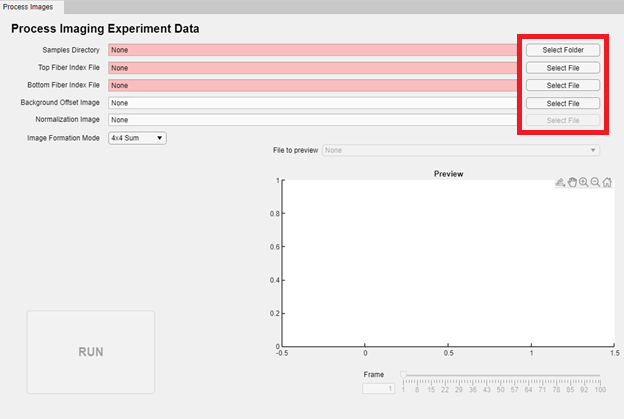

Supplement: Supplementary file 14 — Supplementary Software [file 41467_2024_48987_MOESM14_ESM.zip › Supplementary Code/doc/1.png]

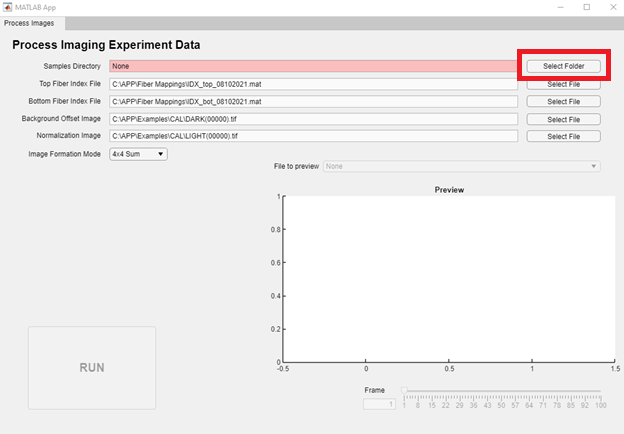

Supplement: Supplementary file 14 — Supplementary Software [file 41467_2024_48987_MOESM14_ESM.zip › Supplementary Code/doc/2.png]

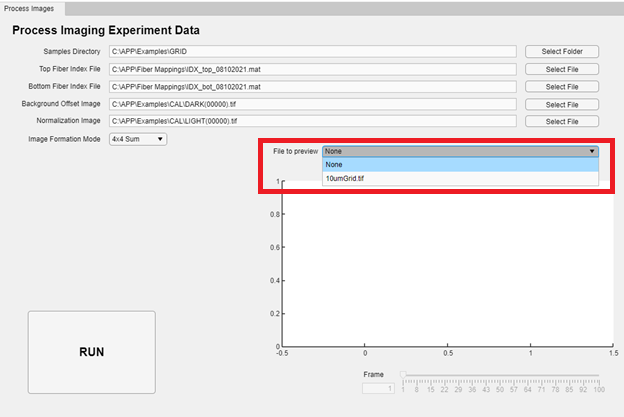

Supplement: Supplementary file 14 — Supplementary Software [file 41467_2024_48987_MOESM14_ESM.zip › Supplementary Code/doc/3.png]

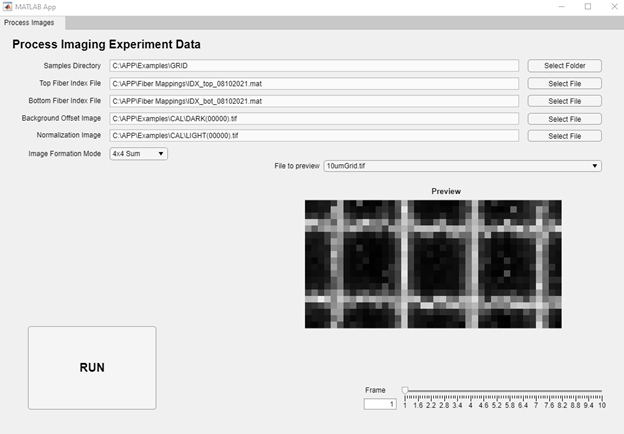

Supplement: Supplementary file 14 — Supplementary Software [file 41467_2024_48987_MOESM14_ESM.zip › Supplementary Code/doc/4.png]

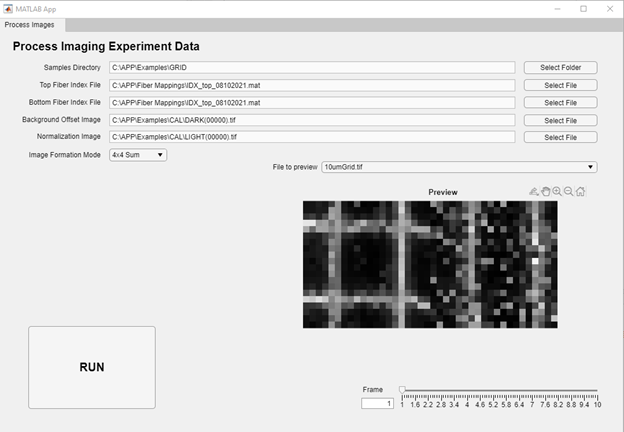

Supplement: Supplementary file 14 — Supplementary Software [file 41467_2024_48987_MOESM14_ESM.zip › Supplementary Code/doc/5.png]

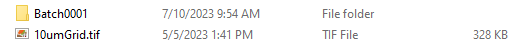

Supplement: Supplementary file 14 — Supplementary Software [file 41467_2024_48987_MOESM14_ESM.zip › Supplementary Code/doc/6.png]

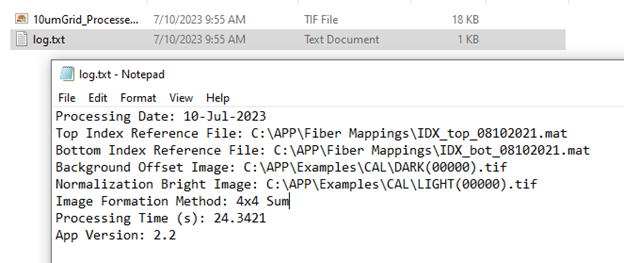

Supplement: Supplementary file 14 — Supplementary Software [file 41467_2024_48987_MOESM14_ESM.zip › Supplementary Code/doc/7.png]

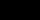

Supplement: Supplementary file 14 — Supplementary Software [file 41467_2024_48987_MOESM14_ESM.zip › Supplementary Code/Examples/Batch0001/grid(00000)_Processed.tif]

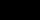

Supplement: Supplementary file 14 — Supplementary Software [file 41467_2024_48987_MOESM14_ESM.zip › Supplementary Code/Examples/Batch0001/group7element5(00000)_Processed.tif]

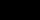

Supplement: Supplementary file 14 — Supplementary Software [file 41467_2024_48987_MOESM14_ESM.zip › Supplementary Code/Examples/Batch0001/star(00000)_Processed.tif]

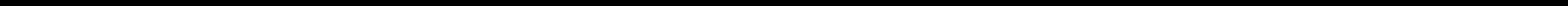

Supplement: Supplementary file 14 — Supplementary Software [file 41467_2024_48987_MOESM14_ESM.zip › Supplementary Code/Examples/CAL/DARK(00000).tif]

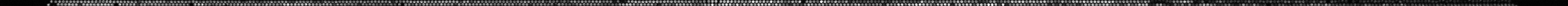

Supplement: Supplementary file 14 — Supplementary Software [file 41467_2024_48987_MOESM14_ESM.zip › Supplementary Code/Examples/CAL/LIGHT(00000).tif]

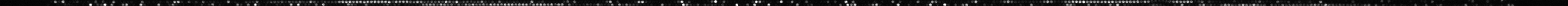

Supplement: Supplementary file 14 — Supplementary Software [file 41467_2024_48987_MOESM14_ESM.zip › Supplementary Code/Examples/grid(00000).tif]

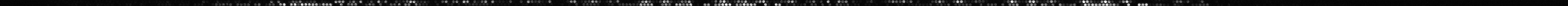

Supplement: Supplementary file 14 — Supplementary Software [file 41467_2024_48987_MOESM14_ESM.zip › Supplementary Code/Examples/group7element5(00000).tif]

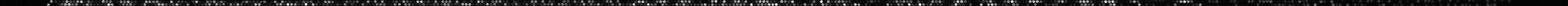

Supplement: Supplementary file 14 — Supplementary Software [file 41467_2024_48987_MOESM14_ESM.zip › Supplementary Code/Examples/star(00000).tif]
