# Supplementary material for: High-speed optical imaging with sCMOS pixel reassignment
Source: Nat Commun. 2024 May 30;15:4598. doi: 10.1038/s41467-024-48987-7 (PMC11139943; doi:10.1038/s41467-024-48987-7)
Supplement: Supplementary file 15 — Reporting Summary [file 41467_2024_48987_MOESM15_ESM.pdf]

nature portfolio

Corresponding author(s): Shu Jia

Last updated by author(s): May 7, 2024

Reporting Summary

Nature Portfolio wishes to improve the reproducibility of the work that we publish. This form provides structure for consistency and transparency in reporting. For further information on Nature Portfolio policies, see our Editorial Policies and the Editorial Policy Checklist.

Statistics

For all statistical analyses, confirm that the following items are present in the figure legend, table legend, main text, or Methods section.

n/a	Confirmed	
x	☐	The exact sample size (n) for each experimental group/condition, given as a discrete number and unit of measurement
☐	x	A statement on whether measurements were taken from distinct samples or whether the same sample was measured repeatedly
x	☐	The statistical test(s) used AND whether they are one- or two-sided<br>*Only common tests should be described solely by name; describe more complex techniques in the Methods section.*
x	☐	A description of all covariates tested
x	☐	A description of any assumptions or corrections, such as tests of normality and adjustment for multiple comparisons
x	☐	A full description of the statistical parameters including central tendency (e.g. means) or other basic estimates (e.g. regression coefficient) AND variation (e.g. standard deviation) or associated estimates of uncertainty (e.g. confidence intervals)
x	☐	For null hypothesis testing, the test statistic (e.g. F, t, r) with confidence intervals, effect sizes, degrees of freedom and P value noted<br>*Give P values as exact values whenever suitable.*
x	☐	For Bayesian analysis, information on the choice of priors and Markov chain Monte Carlo settings
x	☐	For hierarchical and complex designs, identification of the appropriate level for tests and full reporting of outcomes
x	☐	Estimates of effect sizes (e.g. Cohen's d, Pearson's r), indicating how they were calculated

Our web collection on statistics for biologists contains articles on many of the points above.

Software and code

Policy information about availability of computer code

Data collection	All image data was collected using Hamamatsu HCImage 4.8.3.4
Data analysis	Custom MATLAB 2019a software was written for all sHAPR postprocessing, and was tested to be compatible with MATLAB 2019a and 2021a. Software is available at https://github.com/ShuJiaLab/sHAPR. Fiji ImageJ for image colormapping, labeling, and display. OriginPro 2023 was used for plotting in Figure 1. Fiji ImageJ with Trackmate v7.11.1 and StarDist detector was used for flow cytometry tracking. Molecular Devices pClamp 10.6 was used to analyze cardiomyocyte traces. Additional MATLAB 2021a codes were used for neuron and cardiomyocyte analysis and used to generate figures.

For manuscripts utilizing custom algorithms or software that are central to the research but not yet described in published literature, software must be made available to editors and reviewers. We strongly encourage code deposition in a community repository (e.g. GitHub). See the Nature Portfolio guidelines for submitting code & software for further information.

Data

Policy information about availability of data

All manuscripts must include a data availability statement. This statement should provide the following information, where applicable:

- Accession codes, unique identifiers, or web links for publicly available datasets
- A description of any restrictions on data availability
- For clinical datasets or third party data, please ensure that the statement adheres to our policy

Calibration target datasets shown in the current study are included in the Supplementary Software as example data. Additional imaging datasets from the current

study are available under restricted access for the reason of large files sizes, access can be obtained from the corresponding author upon request. Requests will be fulfilled within two weeks. Source data are provided with this paper.

Research involving human participants, their data, or biological material

Policy information about studies with human participants or human data. See also policy information about sex, gender (identity/presentation), and sexual orientation and race, ethnicity and racism.

Reporting on sex and gender	*Use the terms sex (biological attribute) and gender (shaped by social and cultural circumstances) carefully in order to avoid confusing both terms. Indicate if findings apply to only one sex or gender; describe whether sex and gender were considered in study design; whether sex and/or gender was determined based on self-reporting or assigned and methods used. Provide in the source data disaggregated sex and gender data, where this information has been collected, and if consent has been obtained for sharing of individual-level data; provide overall numbers in this Reporting Summary. Please state if this information has not been collected. Report sex- and gender-based analyses where performed, justify reasons for lack of sex- and gender-based analysis.*
Reporting on race, ethnicity, or other socially relevant groupings	*Please specify the socially constructed or socially relevant categorization variable(s) used in your manuscript and explain why they were used. Please note that such variables should not be used as proxies for other socially constructed/relevant variables (for example, race or ethnicity should not be used as a proxy for socioeconomic status). Provide clear definitions of the relevant terms used, how they were provided (by the participants/respondents, the researchers, or third parties), and the method(s) used to classify people into the different categories (e.g. self-report, census or administrative data, social media data, etc.) Please provide details about how you controlled for confounding variables in your analyses.*
Population characteristics	*Describe the covariate-relevant population characteristics of the human research participants (e.g. age, genotypic information, past and current diagnosis and treatment categories). If you filled out the behavioural & social sciences study design questions and have nothing to add here, write "See above."*
Recruitment	*Describe how participants were recruited. Outline any potential self-selection bias or other biases that may be present and how these are likely to impact results.*
Ethics oversight	*Identify the organization(s) that approved the study protocol.*

Note that full information on the approval of the study protocol must also be provided in the manuscript.

Field-specific reporting

Please select the one below that is the best fit for your research. If you are not sure, read the appropriate sections before making your selection.

- [x] Life sciences
- [ ] Behavioural & social sciences
- [ ] Ecological, evolutionary & environmental sciences

For a reference copy of the document with all sections, see nature.com/documents/nr-reporting-summary-flat.pdf

Life sciences study design

All studies must disclose on these points even when the disclosure is negative.

Sample size	Our experiments aim to demonstrate the proof-of-concept sHAPR high speed imaging system. Hence, the sample size was determined from adjusting a wide variety of imaging parameters (alignment, magnification, illumination intensity, positioning) to fine tune the imaging system and ensure a repeatable proof to the viability of our technique.
Data exclusions	Due to the high-speed and remapping of sHAPR, cells could not be pre-screened for activity before recording. Therefore, after reviewing images, neurons that presented no fluorescent activity, presented weakening activity during acquisition, or did not respond to stimulation were excluded. Cardiomyocytes that presented no fluorescence activity or presented weakening activity during acquisition were excluded.
Replication	Target results were replicated reliably in at least two experiments. Microfluidic results were replicated reliably in at least two experiments. Live cell results show multiple examples from >3 experiments.
Randomization	Cell culture samples were used as prepared. Neither specific randomization nor selection procedures were used.
Blinding	Blinding was not necessary, as the aim of the study is to validate the high-speed imaging capabilities of sHAPR.

Reporting for specific materials, systems and methods

We require information from authors about some types of materials, experimental systems and methods used in many studies. Here, indicate whether each material, system or method listed is relevant to your study. If you are not sure if a list item applies to your research, read the appropriate section before selecting a response.

Materials & experimental systems

n/a	Involved in the study	
☒	☐	Antibodies
☐	☒	Eukaryotic cell lines
☒	☐	Palaeontology and archaeology
☐	☒	Animals and other organisms
☒	☐	Clinical data
☒	☐	Dual use research of concern
☒	☐	Plants

Methods

n/a	Involved in the study	
☒	☐	ChIP-seq
☐	☒	Flow cytometry
☒	☐	MRI-based neuroimaging

Eukaryotic cell lines

Policy information about cell lines and Sex and Gender in Research

Cell line source(s)	Hela cells (93021013, Sigma Aldrich)<br>hiPSCs (IMR90-4, WiCell Research Institute)
Authentication	ECACC Cell Lines Undergo Comprehensive Quality Control and Authentication Procedures:<br>•Tested for mycoplasma by culture isolation, Hoechst DNA staining and PCR •Tested for bacteria, yeast and fungi •Species verification by DNA bar-coding and identity verification by DNA profiling •Classical DNA fingerprinting using multi-locus probes is carried out for non-human cell lines<br>WiCellResearch Institute Certificate of analysis:<br>•Karyotype G-T-L Banding •Post-Thaw Viable Cell Recovery •Identity by STR PowerPlex 16 HS System by Promega •Mycoplasma PCR testing •Sterility Native Product Direct Transfer Using FTM and TSB
Mycoplasma contamination	WiCell Research Institute performed PCR mycoplasma testing. ECACC routinely tests all manufactured cell banks for mycoplasma. However, after receiving the cells, cell lines were not tested for mycoplasma contamination.
Commonly misidentified lines<br>(See ICLAC register)	*Name any commonly misidentified cell lines used in the study and provide a rationale for their use.*

Animals and other research organisms

Policy information about studies involving animals; ARRIVE guidelines recommended for reporting animal research, and Sex and Gender in Research

Laboratory animals	18.5-day-old embryos were collected from timed-pregnant Sprague Dawley rats (Charles River Laborato-ries)
Wild animals	None
Reporting on sex	Sex was not considered in this study
Field-collected samples	None
Ethics oversight	Animal care and use were conducted following National Institutes of Health guidelines, and procedures were approved by the Institutional Animal Care and Use Committee at Emory University.

Note that full information on the approval of the study protocol must also be provided in the manuscript.

Plants

Seed stocks	n/a
Novel plant genotypes	n/a
Authentication	n/a

Flow Cytometry

Plots

Confirm that:

- [ ] The axis labels state the marker and fluorochrome used (e.g. CD4-FITC).
- [ ] The axis scales are clearly visible. Include numbers along axes only for bottom left plot of group (a 'group' is an analysis of identical markers).
- [ ] All plots are contour plots with outliers or pseudocolor plots.
- [x] A numerical value for number of cells or percentage (with statistics) is provided.

Methodology

Sample preparation	HeLa cells were stained using Syto-16, washed with HBSS, and detached from the culture dish using trypsin-EDTA. Cells were then fixed using 4% PFA fixation buffer, concentrated using a centrifuge, and suspended into 3mL of PBS. After an additional washing step, cells were stored in 3mL of PBS as the final solution flowed through the microfluidic chip. Full details are available in the Methods section of the main text.
Instrument	Cells were flowed through a PMMA microfluidic chip (Fluidic 386, Chipshop) using a syringe pump (Pump 11 Elite, Harvard Apparatus). Image data was collected using the sHAPR system. Full details are available in the Methods section of the main text.
Software	Image data was collected using HCImage live 4.8.3.4 and post-processed using the custom sHAPR pipeline available at https://github.com/ShuJiaLab/sHAPR. Resulting image data was further analyzed using Fiji ImageJ with the Trackmate plugin and the StarDist detector.
Cell population abundance	All cells were from the same population; study design was to validate high-speed imaging capability.
Gating strategy	No gating strategy was used.

- [ ] Tick this box to confirm that a figure exemplifying the gating strategy is provided in the Supplementary Information.
